# Transinfected *Wolbachia* strains induce a complex of cytoplasmic incompatibility phenotypes: Roles of CI factor genes

**DOI:** 10.1111/1758-2229.13169

**Published:** 2023-05-16

**Authors:** Jing Li, Bei Dong, Yong Zhong, Zheng‐Xi Li

**Affiliations:** ^1^ Department of Entomology and Key Laboratory of Pest Monitoring and Green Management, MOA, College of Plant Protection China Agricultural University Beijing China; ^2^ Jinan Academy of Agricultural Sciences Jinan China; ^3^ Pingxiang Customs Comprehensive Technical Service Center Pingxiang China

## Abstract

*Wolbachia* can modulate the reproductive development of their hosts in multiple modes, and cytoplasmic incompatibility (CI) is the most well‐studied phenotype. The whitefly *Bemisia tabaci* is highly receptive to different *Wolbachia* strains: *w*Ccep strain from the rice moth *Corcyra cephalonica* and *w*Mel strain from the fruit fly *Drosophila melanogaster* could successfully establish and induce CI in transinfected whiteflies. Nevertheless, it is unknown what will happen when these two exogenous *Wolbachia* strains are co‐transinfected into a new host. Here, we artificially transinferred *w*Ccep and *w*Mel into the whitefly and established double‐ and singly‐transinfected *B*. *tabaci* isofemale lines. Reciprocal crossing experiments showed that *w*Ccep and *w*Mel induced a complex of CI phenotypes in the recipient host, including unidirectional and bidirectional CI. We next sequenced the whole genome of *w*Ccep and performed a comparative analysis of the CI factor genes between *w*Ccep and *w*Mel, indicating that their *cif* genes were phylogenetically and structurally divergent, which can explain the crossing results. The amino acid sequence identity and structural features of Cif proteins may be useful parameters for predicting their function. Structural comparisons between CifA and CifB provide valuable clues for explaining the induction or rescue of CI observed in crossing experiments between transinfected hosts.

## INTRODUCTION

*Wolbachia* as maternally inherited obligate intracellular endosymbionts are widespread in terrestrial arthropods (Pannebakker et al., 2007; Saridaki & Bourtzis, 2010; Zug & Hammerstein, 2012). *Wolbachia* induce multiple reproductive manipulations in their hosts (Ant et al., 2018; Gong et al., 2020; Kambris et al., 2009; O'Neill et al., 1997; Pan et al., 2012; Zug & Hammerstein, 2015), which can be used for biocontrol of crop pests and blocking virus transmission (Bourtzis, 2008; Rio et al., 2006; Sinkins & Gould, 2006; Zabalou et al., 2004).

Cytoplasmic incompatibility (CI) is the most widely studied phenotype induced by *Wolbachia*: uninfected females are sterilised by males infected with a *Wolbachia* strain (unidirectional CI), but the females infected with the same strain will be rescued (Werren et al., 2008). Insects infected with different *Wolbachia* strains may display unidirectional or bidirectional CI. Embryonic lethality induced by either strain in males cannot be rescued by the other strain in females (bidirectional CI), forming a reproductive barrier between insect hosts (Brucker & Bordenstein, 2012).

The CI factors (Cifs), including *cif*A and *cif*B, are involved in CI induction and rescue in host insects (Beckmann et al., 2017; Chen et al., 2019; LePage et al., 2017; Shropshire et al., 2018). Transgenic expression of both *cif*A and *cif*B genes, or *cif*B alone, in the germline of male hosts induced sterility that is highly similar to CI induced by *Wolbachia* (Adams et al., 2021; Beckmann et al., 2017; LePage et al., 2017; Shropshire & Bordenstein, 2019; Sun et al., 2022). This embryonic lethality could be rescued by crossing transgenic males with *Wolbachia*‐infected females or those with a transgenic *cif*A gene expressed in the germline (Chen et al., 2019; Shropshire et al., 2018). Recent studies have found that the *cif* genes from bidirectional CI‐inducing *Wolbachia* pairs were highly divergent, with only 29%–68% amino acid identity (LePage et al., 2017). These results were supported by transgenic research using *cif* genes of *w*Mel, *w*Ri, and *w*Rec from *D. melanogaster*, *D*. *simulans*, and *D*. *recens* (Shropshire et al., 2021). The *cifA* and *cifB* genes are co‐divergent (Bonneau et al., 2018; LePage et al., 2017; Lindsey et al., 2018), and have been proposed to account at least in part for bidirectional incompatibility probably by modulating CifA‐CifB binding (Beckmann et al., 2017; Chen et al., 2019).

*Wolbachia* co‐infections are common in arthropods (Funkhouser‐Jones et al., 2015; Łukasik et al., 2013; Machtelinckx et al., 2012; Moutailler et al., 2016; Nguyen et al., 2017; Wamwiri et al., 2013; Zhang et al., 2016; Zytynska & Weisser, 2016), although phenotypic effects of co‐infections are multifarious. Some studies found an enhanced effect or a novel phenotype in co‐infections (Jamnongluk et al., 2002; Kondo et al., 2002; Mouton et al., 2004). For example, in *Cadra cautella*, dual infection with Supergroup A and B *Wolbachia* strains induced complete CI, whereas in *Ephestia kuehniella*, single infection with Supergroup A exhibited only partial CI (Sasaki et al., 2005). In the two‐spotted spider mite *Tetranychus urticae*, *Wolbachia* infection alone induced weak CI, while double‐infected males with *Wolbachia* and *Cardinium* caused strong CI (Xie et al., 2016). In natural populations of the butterfly *Eurema hecabe*, individuals singly infected with *w*HecCI exhibited strong CI, but double‐infected individuals with *w*HecCI and *w*HecFem displayed a phenotype of feminization (Narita et al., 2007).

*Bemisia tabaci* (Hemiptera: Aleyrodidae) is a polyphagous agricultural pest feeds on more than 60 plant species and is a devastating pest insect worldwide. The unfertilised eggs of *B. tabaci*, a haplodiploid species, develop into male offspring, and thus CI can induce a male‐biased offspring sex ratio (Hu & Li, 2015). Our previous studies have shown that it is highly receptive to *Wolbachia* infection: the *Wolbachia w*Ccep strain from the rice moth *Corcyra cephalonica* and the *w*Mel strain from the fruit fly *D*. *melanogaster* could successfully establish and induce CI in *B. tabaci*, which has implications for biological control of pest insects based on *Wolbachia*‐induced CI. Here, we established a stable double‐transinfected (DT) isofemale line of *B. tabaci* with *w*Ccep and *w*Mel. Our purpose was to use this model to examine the CI phenotypes induced by co‐infecting *Wolbachia* strains, and further explore the molecular mechanism underlying CI induction. Our results found that DT *Wolbachia* strains can induce a complex of CI phenotypes, including unidirectional and bidirectional CI, which is consistent with genomic analysis based on *cif* genes. Furthermore, our data provide valuable clues for explaining the induction or rescue of CI observed in crossing experiments using host insects transinfected with different *Wolbachia* strains.

## RESULTS

### Establishment of transinfected *B. tabaci* isofemale lines and trans‐generational maintenance

**FIGURE 1:**
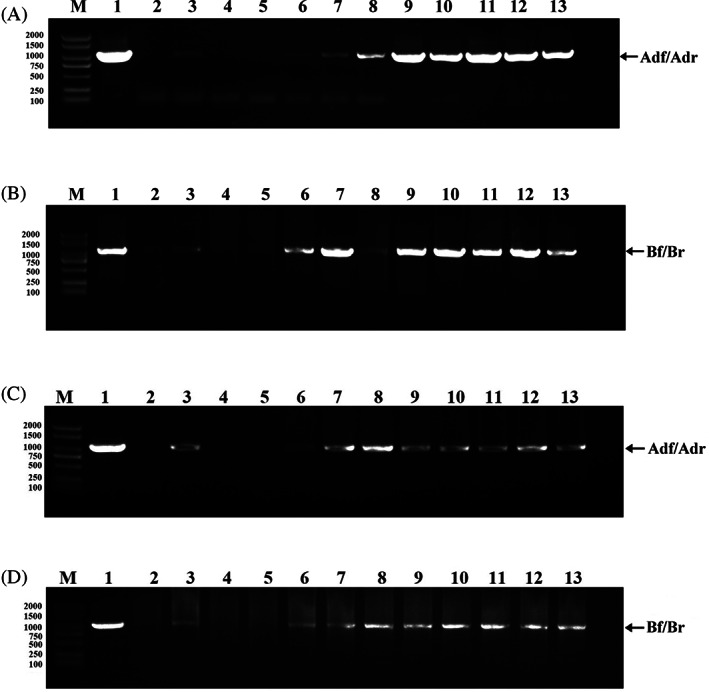
PCR detection of transinfected *B. tabaci* whitefly isolines from G_0_ to G_10_ using the primers targeting *ftsZ* of Supergroup A and B *Wolbachia*. (A) *ftsZ* primer pair Adf/Adr targeting Supergroup A *Wolbachia* in double‐transinfected (DT) individuals. Lane 1: positive control (*D. malanogaster* adult infected with *w*Mel); lane 2: negative control (wild‐type *B. tabaci*); lane 3–13: *B. tabaci* individuals collected from G_0_ to G_10_, respectively. (B) *ftsZ* primer pair Bf/Br targeting Supergroup B *Wolbachia* in DT individuals. Lane 1: positive control (*C. cephalonica* adult infected with *w*Ccep); lane 2: negative control (wild‐type *B. tabaci*); lane 3–13: *B. tabaci* individuals collected from G_0_ to G_10_, respectively. (C) *ftsZ* primer pair Adf/Adr targeting Supergroup A *Wolbachia* in *w*Mel singly‐transinfected (ST) individuals. Lane 1: positive control; lane 2: negative control; lane 3–13: *B. tabaci* individuals collected from G_0_ to G_10_, respectively. (D) *ftsZ* primer pair Bf/Br targeting Supergroup B *Wolbachia* in *w*Ccep ST individuals. Lane 1: positive control; lane 2: negative control; lane 3–13: *B. tabaci* individuals collected from G_0_ to G_10_, respectively. M, DNA molecular marker.

Fifteen (five DT and 10 singly‐transinfected [ST]) transinfected *B. tabaci* isofemale lines were established, which were trans‐generationally maintained for 10 generations from G_1_ to G_10_. As detected by polymerase chain reaction (PCR) using the *ftsZ* primers specific to Supergroup A and B *Wolbachia*, the individuals collected from G_3_ to G_10_ tested positive for both *w*Mel and *w*Ccep. In addition, a strong signal for *w*Mel and *w*Ccep was detected in the individuals from G_6_ and G_10_ in a stable manner, although no signal was detected in the individuals from G_1_ and G_2_ (Figure 1A,B). In ST isolines, *w*Mel or *w*Ccep was detected in the individuals collected from G_3_ to G_10_ (Figure 1C,D).

### Crossing experiments and CI phenotypes

**FIGURE 2:**
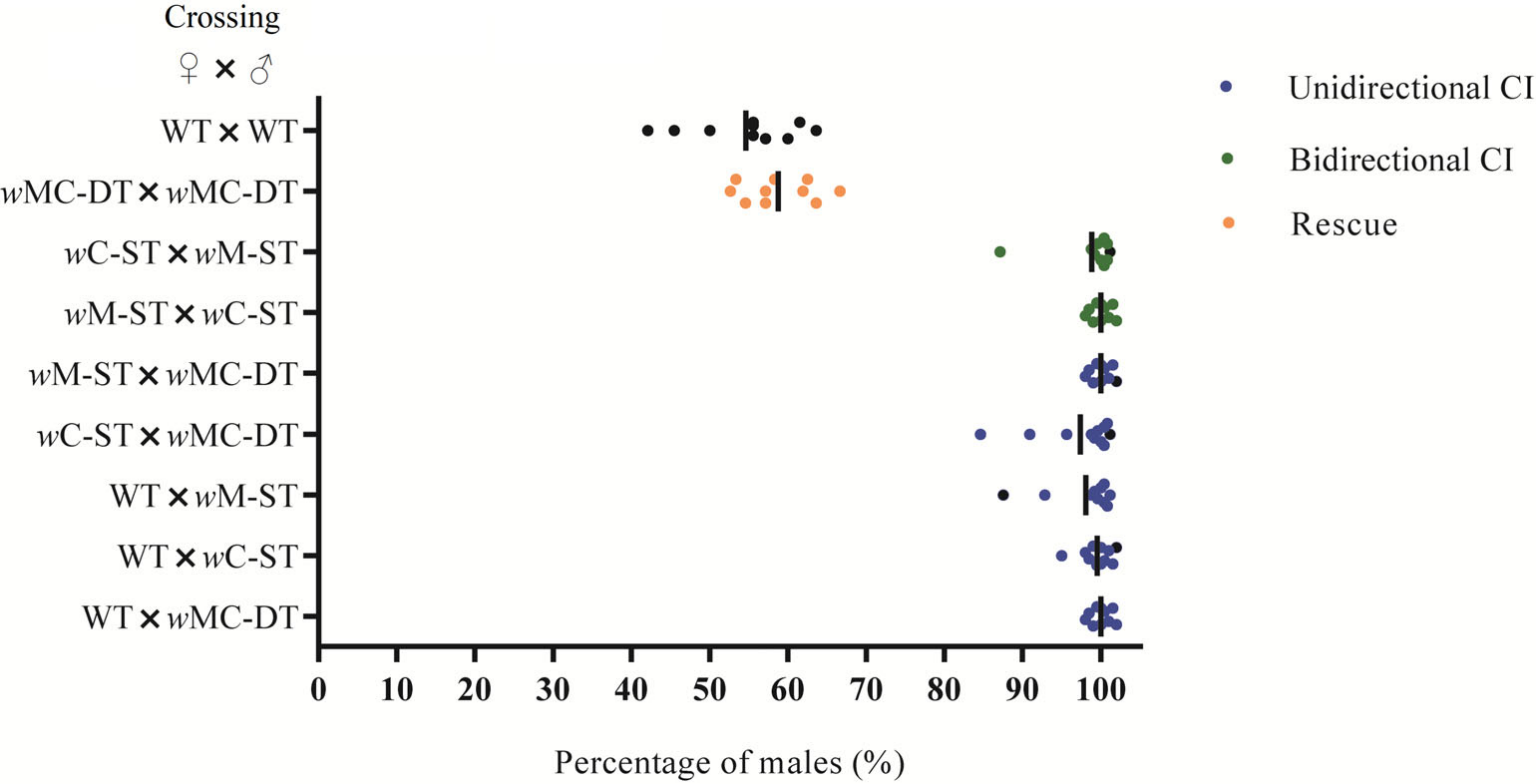
Crossing experiments between double‐transinfected (DT), singly‐transinfected (ST) and wild‐type *B. tabaci* whiteflies. A total of nine groups of crossings are performed. WT, wild‐type; *w*MC‐DT, DT with *w*Mel and *w*Ccep; *w*C‐ST, ST with *w*Ccep; *w*M‐ST, singly‐transinfected with *w*Mel. The means are indicated by vertical lines.

We performed a total of nine groups of crossing experiments. The results showed that there was no significant difference in the number of offspring per female among different crossing groups (Student–Newman–Keuls [SNK], *F* = 0.686, *p* = 0.703), while the percentages of males produced among crossing groups were significantly different (SNK, *F* = 241.295, *p* < 0.001; Table 1). Specifically, there was a very high percentage of males (97.4%–100%) in *w*C‐ST♀ × *w*M‐ST♂, *w*M‐ST♀ × *w*C‐ST♂, *w*M‐ST♀ × *w*MC‐DT♂, *w*C‐ST♀ × *w*MC‐DT♂, WT♀ × *w*M‐ST♂, WT♀ × *w*C‐ST♂ and WT♀ × *w*MC‐DT♂, representing a high level of CI. Among them, the two crossing groups *w*C‐ST♀ × *w*M‐ST♂ and *w*M‐ST♀ × *w*C‐ST♂ showed typical bidirectional CI phenotype, in which the male and female were ST with different *Wolbachia* strains and the reproductive anomaly caused by CI induced by one strain cannot be rescued by the other strain. In contrast, the crossing groups WT♀ × *w*M‐ST♂, WT♀ × *w*C‐ST♂ and WT♀ × *w*MC‐DT♂ exhibited unidirectional CI phenotype (Figure 2).

**TABLE 1:** Crossing experiments between double‐transinfected, singly‐transinfected and wild‐type *B. tabaci* whiteflies*.*

Crossing (♀ × ♂)	No. of crosses	No. of progenies	Percentage of males (%)
WT × WT	10	14.6 ± 3.14a	54.7 ± 3.98c
*w*MC‐DT × *w*MC‐DT	10	13.5 ± 2.52a	58.8 ± 2.72b
*w*C‐ST × *w*M‐ST	10	15.4 ± 4.33a	98.7 ± 2.36a
*w*M‐ST × *w*C‐ST	10	14.8 ± 3.45a	100 ± 0.00a
*w*M‐ST × *w*MC‐DT	10	11.0 ± 2.75a	100 ± 0.00a
*w*C‐ST × *w*MC‐DT	11	13.9 ± 3.12a	97.4 ± 2.96a
WT × *w*M‐ST	13	12.5 ± 3.59a	99.6 ± 0.80a
WT × *w*C‐ST	10	14.5 ± 2.16a	98.0 ± 2.50a
WT × *w*MC‐DT	11	13.1 ± 2.55a	100 ± 0.00a

*Note*: Data are means ± SE of three biological replicates. Different lowercase letters in the same column indicate significant difference based on One‐way ANOVA followed by Student–Newman–Keuls test (*p* < 0.05).

### Genome sequence of *w*Ccep

The complete circular genome of *w*Ccep was assembled using long‐read PacBio sequencing at 500× median coverage (GenBank acc.no. CP087954; Figure S1). The total length of *w*Ccep genome was 1,359,904 bp, with an average GC content of 34.0%, within the range of a typical *Wolbachia* genome (31.7%–38.3%). Annotation of the genome identified 1278 CDSs, 1190 protein‐coding genes, 34 tRNA genes that can transfer all 20 amino acids, three rRNA (5S, 16S and 23S rRNA) genes, and one sRNA gene (Table 2). Phylogenetic analysis based on *Wolbachia* genome sequences confirmed that *w*Ccep belonged to Supergroup B (Figure S2; Table S1).

**TABLE 2:** Genome statistics of *Wolbachia w*Ccep strain (host: *C. cephalonica*).

	Number
Genome size	1,381,880
Total no. of nucleotides (bp)	1,359,904
GC content (%)	34.0
No. of proteins	1190
No. of CDSs	1278
No. of tRNAs	34
No. of rRNAs	3
No. of sRNA	1
Average gene length	1047.7

### Identification of *cif* genes in ***w*** Ccep and phylogenetic analysis

**FIGURE 3:**
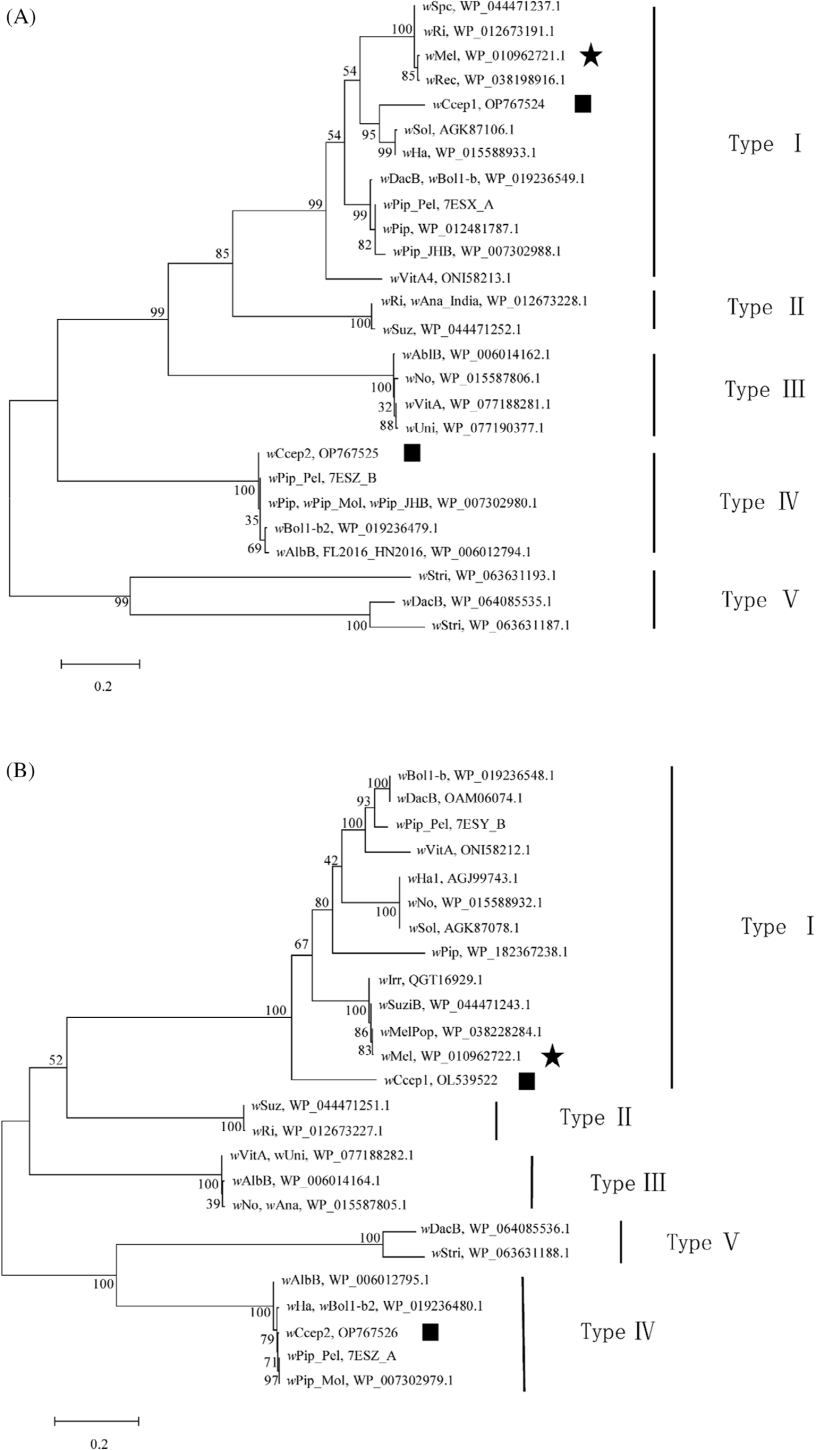
Phylogenetic analysis of CIF proteins using maximum likelihood method on MEGA 7.1. The CIF proteins used in the analysis are listed in Table S2. (A) CifA. (B) CifB. The CIF proteins of *w*Ccep are indicated by solid squares, and those of *w*Mel by solid stars.

Genomic analysis identified five *cif* genes in the *w*Ccep genome, including *cif*A1 (GenBank acc. no. OP767524), *cifB*1 (GenBank acc. no. OL539522), *cif*A2 (GenBank acc. no. OP767525), *cif*B2 (GenBank acc. no. OP767526) and an incomplete *cif*A. Phylogenetic analysis using the four Cif proteins of *w*Ccep and reference Cifs showed that CifA1^*w*Ccep^ and CifA^*w*Mel^ were clustered in a clade belonging to Type I CifA, and CifA2^*w*Ccep^ was clustered in a clade belonging to CifA Type IV (Martinez et al., 2021; Figure 3A). CifB1^*w*Ccep^ and CifB^*w*Mel^ were clustered in a clade of Type I CifB, while CifB2^*w*Ccep^ was grouped in Type IV CifB (Figure 3B).

### Structural comparison of CIF proteins between *w*Ccep and *w*Mel

**FIGURE 4:**
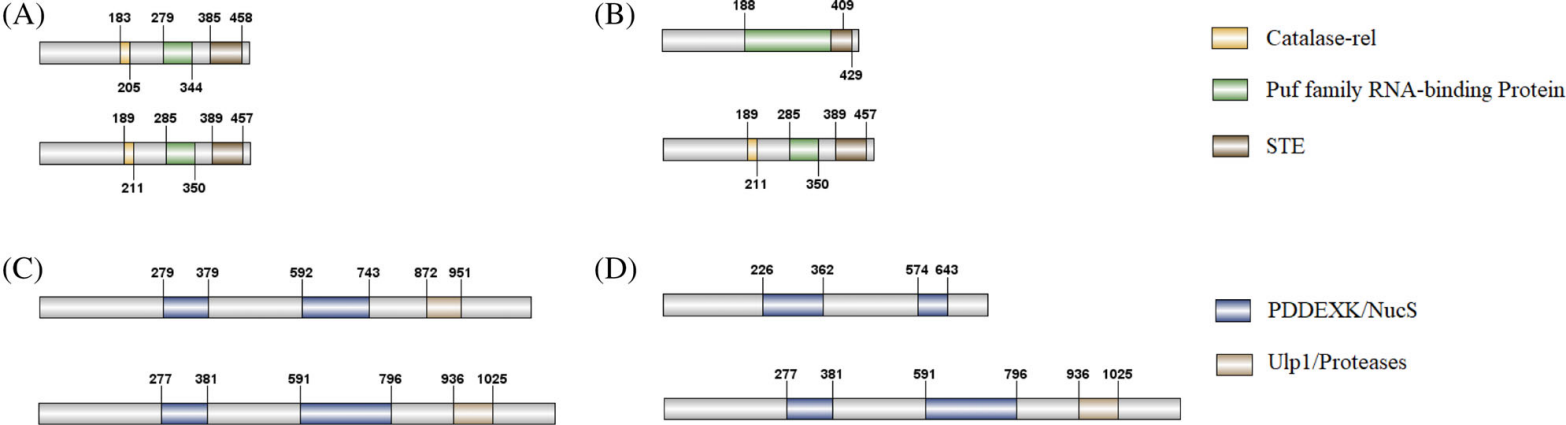
Comparisons of the organisations and domains of CIF proteins between *w*Mel and *w*Ccep. (A) CifA I^*w*Ccep^ and CifA I^*w*Mel^; (B) CifA IV^*w*Ccep^ and CifA I^*w*Mel^; (C) CifB I^*w*Ccep^ and CifB I^*w*Mel^; (D) CifB IV^*w*Ccep^ and CifB I^*w*Mel^. The domains include a putative catalase‐related (catalase‐rel) domain (gold), a domain sharing homology with Puf family RNA‐binding proteins (green), a sterile‐like transcription factor (STE) domain (brown), a Ulp1 deubiquitinase module (indigo blue), and a PDDEXK nuclease domain (dark brown). The numbers indicate the start and stop positions of each domain.

Structural prediction revealed the putative domains of CIF proteins. There was variation only in the length of domains between CifA I^*w*Ccep^ and CifA I^*w*Mel^, and between CifB I^*w*Ccep^ and CifB I^*w*Mel^ (Figure 4A,C), while substantial structural differentiation was observed between CifA I and CifA IV, and between CifB I and CifB IV (Figure 4B,D). CifA I contained a putative catalase‐related (catalase‐rel) domain, a domain that shares homology with Puf family RNA‐binding proteins and a sterile‐like transcription factor (STE) domain (Figure 4A), whereas CifA IV contained a longer Puf domain but no catalase‐rel domain (Figure 4B). In contrast, CifB I contained two PD‐(D/E)XK superfamily of nucleases domains and a Ulp1 ubiquitin protease module with deubiquitylase (DUB) activity (Figure 4C), but CifB IV contained no Ulp1 domain (Figure 4D).

### Comparison of the tertiary structures of CIF proteins between *w*Ccep and *w*Mel

**FIGURE 5:**
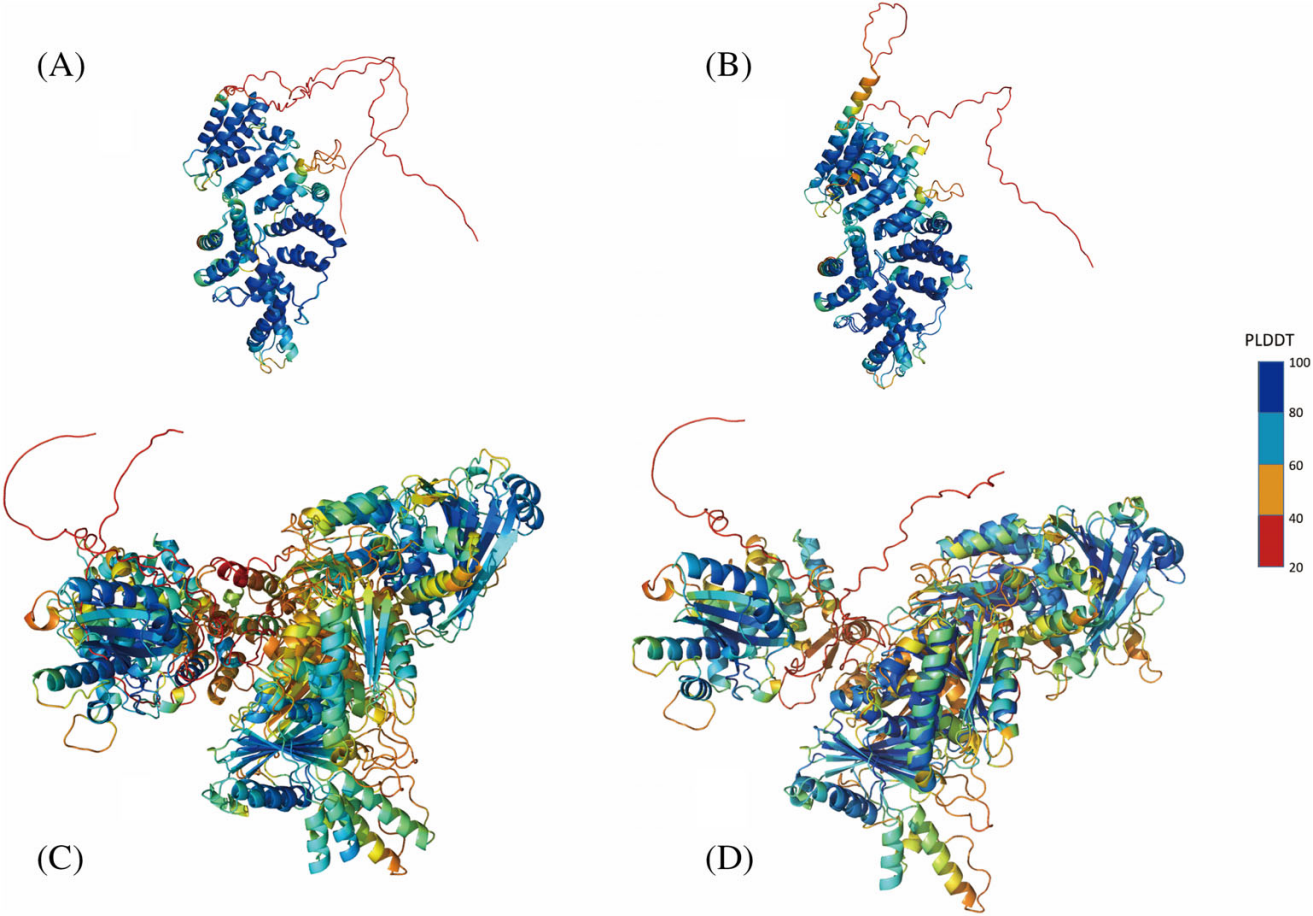
Overlapping CIF proteins of *w*Mel and *w*Ccep generated by Alphafold programme. (A) Overlapped CifA I^*w*Ccep^ and CifA I^*w*Mel^; (B) Overlapped CifA IV^*w*Ccep^ and CifA I^*w*Mel^; (C) Overlapped CifB I^*w*Ccep^ and CifB I^*w*Mel^; (D) Overlapped CifB IV^*w*Ccep^ and CifB I^*w*Mel^. The domains include a putative catalase‐related (catalase‐rel) domain (grey), a domain sharing homology with Puf family RNA‐binding proteins (green), a sterile‐like transcription factor (STE) domain (brown), a Ulp1 deubiquitinase module (gold), and a PDDEXK nuclease domain (light grey).

The tertiary structures of CIF proteins were generated by AlphaFold. The confidence of the structures was evaluated by Local Distance Difference Test (lDDT; Figure S3). The results showed that the lDDT scores of all of the six Cif proteins of *w*Ccep and *w*Mel ranged between 68.4 and 84.0, suggesting a confident structural prediction based on the full‐length sequences using the AlphaFold programme, except for CifB I^*w*Mel^ containing a DUB domain with a low predicted mass (Table 3; Figure S4). Only two PD‐(D/E)XK‐nuclease domains of CifB were analysed because they were shared across all types of Cif proteins (Shropshire et al., 2022), and it was difficult to make a direct determination of the structure of CifB^*w*Mel^ because its expression in *Escherichia coli* was poor (Wang et al., 2022). To evaluate the similarity between the tertiary structures of CIF proteins, the template modelling (TM) scores were calculated (Xu & Zhang, 2010). The results showed that the TM score between CifA I^*w*Ccep^ and CifA I^*w*Mel^ was 0.86, while they shared an amino acid sequence identity (AA identity) of 67.1%; the TM score between CifB I^*w*Ccep^ and CifB I^*w*Mel^ was 0.63, while shared an AA identity of 57.3%. Although CifA IV^*w*Ccep^ and CifA I^*w*Mel^ shared an AA identity of only 26.3%, the TM score between them was 0.76. The TM score between CifB IV^*w*Ccep^ and CifB I^*w*Mel^ was 0.66, but they shared an AA identity of only 20.3% (Table 4; Figure 5).

**TABLE 3:** Alphafold structural confidence of CIF proteins.

Protein	Length	Mean lDDT ± SD^a^
CifA I^*w*Ccep^	475	81.1 ± 0.79
CifA I^*w*Mel^	474	82.0 ± 0.83
CifA IV^*w*Ccep^	445	84.0 ± 1.42
CifB I^*w*Ccep^	1107	71.9 ± 1.90
CifB I^*w*Mel^	1166	68.4 ± 2.47
CifB IV^*w*Ccep^	732	82.1 ± 1.17

^a^ lDDT scores range from 0 to 100: lDDT > 90, high confidence; 90 > lDDT > 70, confident; 70 > lDDT > 50, low confidence, and lDDT < 50, very low confidence.

**TABLE 4:** TM‐scores of CIF proteins*.*

AA identity% TM score	CifA I^*w*Ccep^	CifA I^*w*Mel^	CifA IV^*w*Ccep^	CifB I^*w*Ccep^	CifB I^*w*Mel^	CifB IV^*w*Ccep^
CifA I^*w*Ccep^	–	67.1	–	–	–	–
CifA I^*w*Mel^	0.86	–	26.3	–	–	–
CifA IV^*w*Ccep^	–	0.76	–	–	–	–
CifB I^*w*Ccep^	–	–	–	–	57.3	–
CifB I^*w*Mel^	–	–	–	0.63	–	20.3
CifB IV^*w*Ccep^	–	–	–	–	0.66	–

*Note*: AA identities are shown above the diagonal. TM‐scores are shown below the diagonal. TM‐scores are calculated by the Zhanglab Server and range from 0 to 1: TM > 0.5, a very similar fold; 0.5 > TM > 0.17, significant similarity and TM < 0.17, random structural similarity.

### CifAI^*w*Ccep^ binds CifBI^*w*Ccep^ through a large distinct interface

**FIGURE 6:**
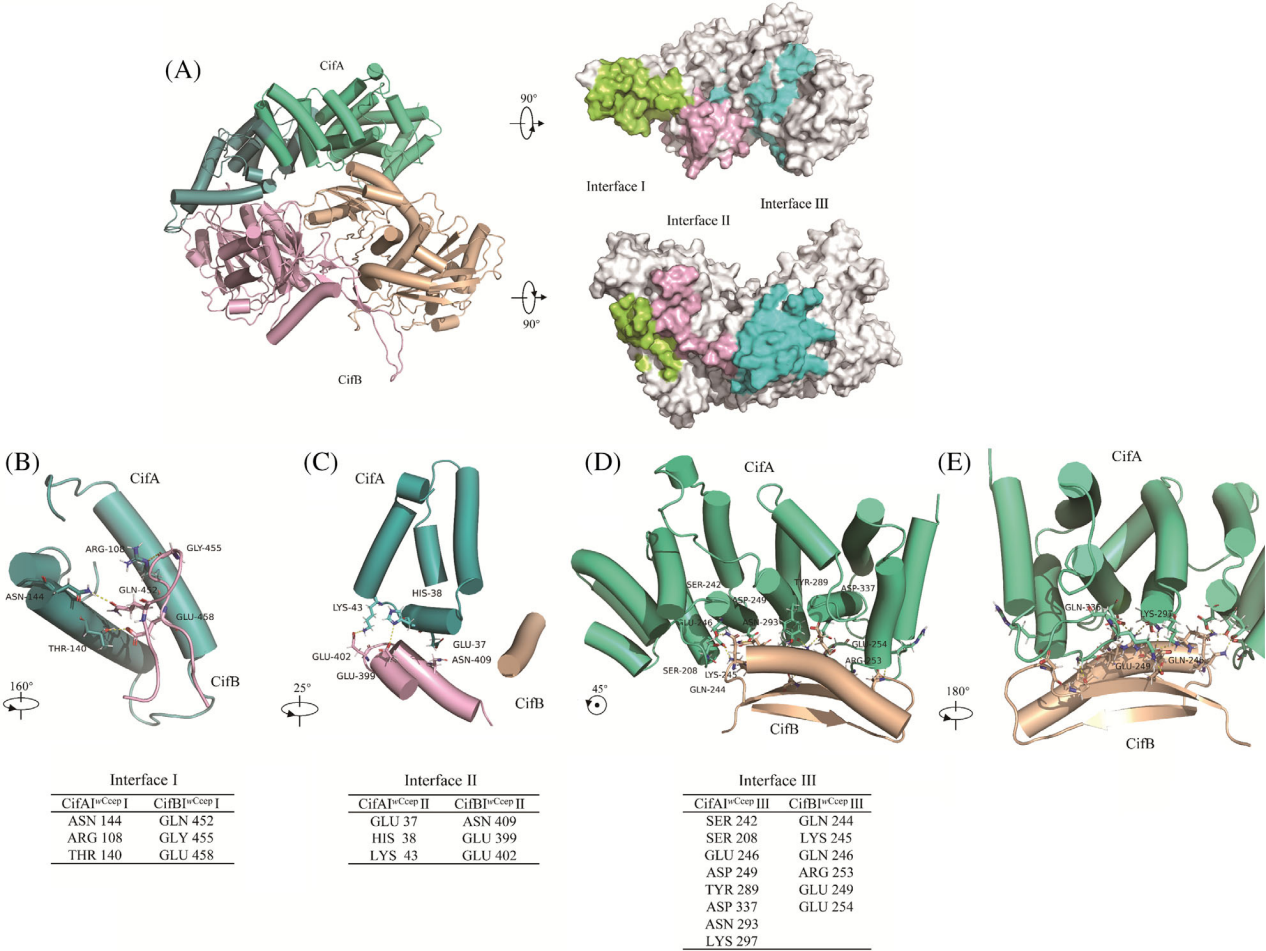
Interaction between CifA and CifB proteins through a large conserved tripartite interface. (A) A structural model of CifA I^*w*Ccep^ in complex with CifB I^*w*Ccep^_2ND_ generated by AlphaFold‐Multimer. CifA I^*w*Ccep^ binds to CifB I^*w*Ccep^_2ND_ through three regions shown in green, pink and cyan for Interface I, II and III, respectively. (B–E) Each interface of the CifA I^*w*Ccep^‐ CifB I^*w*Ccep^_2ND_ complex involves a pair of structural motifs. Representative residues directly involved in the interaction are labelled and tabulated. 2ND, two PD‐(D/E)XK nuclease domains.

A model for the CifA I^*w*Ccep^‐CifB I^*w*Ccep^_2ND_ complex was built by using AlphaFold‐Multimer (Figure 6A). CifB I^*w*Ccep^_2ND_ comprised residues 1 through 752, which was predicted to contain two PD‐(D/E)XK (pseudo) nuclease domains. The obtained end‐to‐end model was further optimised by molecular dynamics (MD) simulations. The trajectories were performed and achieved equilibrium within 100 ns. The root mean square deviation (RMSD) for one of the simulation trajectories was recorded (Figure S5A). The complex also predicted many hydrogen bonds and salt bridges between CifA I^*w*Ccep^ and CifB I^*w*Ccep^_2ND_ at the three interfaces (Figure 6B–E) and only 0.46% of the residues were Ramachandran outliers (Figure S5B), demonstrating that the side chains of the predicted model have been reliably predicted and thus the complex of CifA I^*w*Ccep^‐CifB I^*w*Ccep^_2ND_ is reasonable.

The interface between CifA I^*w*Ccep^ and CifB I^*w*Ccep^_2ND_ was divided into three regions (Figure 6A), and each region mainly involves one pair of structural motifs. In the first region (Interface I), the helices consisting of the residues 38–143 of CifA I^*w*Ccep^ interact with a loop in CifB I^*w*Ccep^_2ND_ (residues 402–425) through a network of hydrogen bonds (Figure 6B). At interface I, three residues (ASN144, ARG108 and THR140) from CifA I^*w*Ccep^ interact with the residues from CifB I^*w*Ccep^_2ND_ (GLN452, GLY455 and GLU458; Figure 6B). Interface II is located between the helices from CifA I^*w*Ccep^ (residues 38–43) and CifB I^*w*Ccep^_2ND_ (residues 399–402), and the residues (GLU37, HIS38 and Lys43) from CifA I^*w*Ccep^ interact with the residues from CifB I^*w*Ccep^_2ND_ (ASN409, GLU399 and GLU402) through a network of four hydrogen bonds and two salt bridges (Figure 6C). Interface III involves CifA I^*w*Ccep^ (residues 208, 242–249, 289–297, 337 and 392–398) and a cross‐cutting helix (residues 244–254, 377–382) in CifB I^*w*Ccep^_2ND_ to stabilise the interaction. Multiple residues interact between CifA I^*w*Ccep^ and CifB I^*w*Ccep^_2ND_ and there are 12 hydrogen bonds and six salt bridges at Interface III (Figure 6D,E).

## DISCUSSION

We established stable double‐ and ST *B. tabaci* isofemale lines with *w*Mel and *w*Ccep. These isolines were used for reciprocal crossing experiments, showing that a complex of CI phenotypes was induced, including unidirectional and bidirectional CI. *B. tabaci* is a haplodiploid species, and it is capable of laying haploid eggs that develop into males (arrhenotokous parthenogenesis) when CI is induced (Hu & Li, 2015; Zhou & Li, 2016). A high male‐biased offspring sex ratio indicates a high CI level. Here, the percentages of males produced in the crossing groups *w*C‐ST♀ × *w*M‐ST♂, *w*M‐ST♀ × *w*C‐ST♂, *w*M‐ST♀ × *w*MC‐DT♂, *w*C‐ST♀ × *w*MC‐DT♂, WT♀ × *w*M‐ST♂, WT♀ × *w*C‐ST♂ and WT♀ × *w*MC‐DT♂ were 100% or nearly 100%, indicating that a complete or nearly complete CI had been induced in these crossings. As expected, no CI phenotype was observed in *w*MC‐DT♀ × *w*MC‐DT♂, which might have been restored through what is usually called “the rescue (resc) function” in the eggs infected with the same *Wolbachia* strains. In *w*C‐ST♀ × *w*M‐ST♂ and *w*M‐ST♀ × *w*C‐ST♂, the male and female were ST with different *Wolbachia* strains and the reproductive anomaly caused by CI induced by one strain cannot be rescued by the other strain, which represents a typical bidirectional CI phenotype. In contrast, the crossing groups WT♀ × *w*M‐ST♂, WT♀ × *w*C‐ST♂ and WT♀ × *w*MC‐DT♂ exhibited unidirectional CI phenotype, in which transinfected males induced CI in wild‐type females.

It has been shown that the Cifs (CifA and CifB) were determinants for CI induction and rescue (Adams et al., 2021; Shropshire & Bordenstein, 2019; Sun et al., 2022), and the expression of CifA in female was sufficient for rescuing CI (Shropshire et al., 2018). We, therefore, sequenced the whole genome of *w*Ccep, identifying two *cif*A and two *cif*B genes. In contrast, the genome of *w*Mel contained only one *cif*A and one *cif*B (Wu et al., 2004). Crossing experiments showed that both *w*Ccep and *w*Mel are CI‐inducing *Wolbachia* strains, and the strength of CI induced by *w*Ccep (WT × *w*C‐ST) was not significantly different from that by *w*Mel (WT × *w*M‐ST; Table 1). It seems that the higher copy number of *cif* genes in *w*Ccep cannot significantly differentiate it from *w*Mel in CI induction. Similarly, the strength of CI induced by DT males is not significantly different from that by ST males, although the CI phenotype induced by double transinfection is more stable than single transinfections.

Previous studies showed that the amino acid similarity between CifA proteins influenced the rescue of CI phenotype. The rescue happened only when the *cif* genes were closely related between different *Wolbachia* strains (Bonneau et al., 2018; LePage et al., 2017; Shropshire et al., 2018). For instance, *w*Ri could rescue *w*Mel‐induced CI in a homozygous cross, because the amino acid sequences of their CifA proteins were nearly identical (99% AA identity) (Shropshire et al., 2021). In contrast, *w*Ha could not rescue CI induced by *w*Mel, and vice versa, because the AA identity between their CifA proteins was only 67% (LePage et al., 2017). In our study, the AA identities between CifA I^*w*Ccep^ and CifA I^*w*Mel^ and between CifB I^*w*Ccep^ and CifB I^*w*Mel^ were 67.1% and 57.3%, respectively. The divergence of Cif proteins between *w*Ccep and *w*Mel can explain the bidirectional CI observed in *w*C‐ST♀ × *w*M‐ST♂ and *w*M‐ST♀ × *w*C‐ST♂, in which CI induced by one strain cannot be rescued by the other. It is noteworthy that the AA identities between CifA IV^*w*Ccep^ and CifA I^*w*Mel^ and between CifB IV^*w*Ccep^ and CifB I^*w*Mel^ were only 26.3% and 20.3%, respectively. The substantial divergence between these Cif proteins may enhance the incompatibility between the two *Wolbachia* strains.

Structural comparisons of Cif proteins between *w*Ccep and *w*Mel revealed that CifA I and CifB I proteins of *w*Ccep and *w*Mel are structurally similar, containing common domains of CIF proteins. However, substantial structural variation was observed in CifA IV and CifB IV of *w*Ccep, with some missing domains (Figure 4). It is still unknown whether they are functionally incompetent or not before further functional studies are carried out. Interestingly, a comparison of the tertiary structures of Cif proteins between *w*Ccep and *w*Mel resulted in a high TM score (0.86) between CifA I^*w*Ccep^ and CifA I^*w*Mel^, whereas a moderate TM score (0.63) was obtained between CifB I^*w*Ccep^ and CifB I^*w*Mel^, even lower than the TM scores between CifA IV^*w*Ccep^ and CifA IV^*w*Mel^ (0.76) and between CifB IV^*w*Ccep^ and CifB IV^*w*Mel^ (0.66). Crossing experiments using transgenic models are needed for functional verification of these *cif* genes.

Previous studies suggested that the interfacial residues of the CifA‐CifB complex that simulated CifA‐CifB binding in the female were essential for the rescue of CI in transgenic *D. melanogaster* (Wang et al., 2022; Xiao et al., 2021). In this study, CifA I^*w*Ccep^‐CifB I^*w*Ccep^_2ND_ complex (except for the DUB domain) built by homology modelling is very similar to the structure of CidA^*w*Mel^‐CidB^*w*Mel^_ND1‐ND2_ complex in which CidA binding does not block the DUB domain of CidB catalytic activity (Beckmann et al., 2017). Both of two complexes form three binding sites in similar positions. Interestingly, the residues involved in the interactions at the three interfaces are very different between the both complexes (Wang et al., 2022; Figure 2C–E), which helps explain their cognate‐specific binding.

In summary, we established stable transinfected *B. tabaci* isofemale lines. Successful establishment of DT isolines demonstrated that *w*Ccep and *w*Mel can co‐exist in the same recipient host, confirming that *B. tabaci* is highly receptive to *Wolbachia* infection. This research model is useful for deciphering the molecular mechanisms underlying CI induction by *Wolbachia*. We performed a series of crossing experiments using these transinfected isolines, and a complex of CI phenotypes were induced. We therefore sequenced the whole genome of *w*Ccep and identified four Cif genes in its genome. Comparative analysis revealed that the CI phenotypes induced by *w*Ccep and *w*Mel can be explained by the divergence between their *cif* genes. Our study confirmed that the amino acid sequence identity is an important parameter for defining CI phenotypes. Nonetheless, structural comparisons in terms of the interfacial residues in the binding regions of the CifA‐CifB complex can provide valuable clues for explaining the induction or rescue of CI observed in crossing experiments using host insects transinfected with different *Wolbachia* strains.

## EXPERIMENTAL PROCEDURES

### Insect rearing and *Wolbachia* isolation

*B*. *tabaci* was reared on the cotton plants in an artificial climate incubator (GXZ‐280C, Jiangnan, China) under a photoperiod of L14:D10 at 28°C and 60%–80% RH. *D. melanogaster* infected with *w*Mel was maintained on Maize–Agarose–Yeast medium (25°C, 60%–70% 100 RH and 14L:10D). The rice moth *C. cephalonica* infected with *w*Ccep was maintained on the maize‐rice bran‐sugar medium (25°C, 65% ± 1 RH and 14L:10D). The *w*Mel and *w*Ccep strains were isolated from the hosts using the Percoll density‐gradient centrifugation method (Zhou & Li, 2016). The purified bacterial extract was detected by PCR using the primers 81F/691R and 81F/522R targeting *wsp* (*Wolbachia* surface protein) of Supergroup A and B *Wolbachia* (*wsp*81F: 5′‐TGG TCCAATAAGTGATGAAGAAAC‐3′, *wsp*522R: 5′‐ACCAGCTTTTGCTTGATA‐3′ and *wsp*691R: 5′‐AAAAATTAAACGCTACTCCA‐3′; Zhou et al., 1998).

### Transinfection and establishment of double‐ and ST isofemale lines of *B. tabaci* with *w*Mel and *w*Ccep *Wolbachia* strains

A volume of 46 nL bacterial suspensions of *w*Ccep and *w*Mel (1:1) in SPG buffer (220 mM sucrose, 4 mM KH2PO4, 9 mM Na2HPO4, 5 mM l‐glutamate, pH 7.4) were injected separately into the fourth‐instar nymph (pseudopupa) of *B. tabaci* placed in a petri dish (Φ 9 cm) covered with 10% agar on the platform of Nanoliter 2000 (World Precision Instruments, Sarasota, Florida, USA). The pupa was then placed in a climate incubator until adult emergence after injection (25°C, 65% ± 1 RH and 14L:10D). The newly emerged adults were separately maintained on potted cotton plants in pairs (♀/♂; G_0_), and the offspring (G_1_) from the pairs tested positive for *Wolbachia* was kept for establishing isofemale lines. For establishing ST isofemale lines, the *w*Ccep strain or *w*Mel strain was used for transinfection following the same procedures.

### Trans‐generational maintenance of transinfected isolines

The established DT and single‐transinfected (ST) *B. tabaci* isofemale lines (G_1_), were trans‐generationally maintained for generations. The offspring of DT and ST whiteflies was detected at each generation for the presence of *w*Ccep and *w*Mel by PCR using the primers targeting the *fts*Z gene of *Wolbachia*. The *w*Mel and *w*Ccep strains, belonging to Supergroup A and B (Hu & Li, 2015), respectively, can be discriminated by their *fts*Z sequences (Werren et al., 1995). Total genomic DNAs were extracted from DT whiteflies using the KAc method as described (Zhong & Li, 2014). The primers for detecting *w*Mel were as followed: Forward Adf: 5′‐CTCAAGCACTAGAAAAGTCG‐3′; reverse Adr: 5′‐TTAGCTCCTTCGCTTACC TG‐3′; for *w*Ccep, Bf: 5′‐CCGATGCTCAAGCGTTAGAG‐3′; Br 5′‐CCACTTAACT CTTTCGTTTG‐3′ (Werren et al., 1995). PCR was performed in a final reaction volume of 25 μL, including 2 μL of gDNA, 0.2 μL of *Taq* DNA polymerase, 1 μL each of 10 μM forward and reverse primers, 2.5 μL of 10 × Easy*Taq* Buffer, and 2.5 μL of 10 μM dNTPs, on an Applied Biosystems Veriti thermal cycler. The PCR cycling programme consisted of 94°C for 2 min, 40 cycles of 94°C for 1 min, 55°C for 1 min and 72°C for 2 min, and a final extension for 10 min at 72°C. PCR products were detected by 1% agarose gel electrophoresis (1 × TAE).

### Crossing experiments

Two‐day‐old adult male and female whiteflies collected from the 6th–8th generation (G_6_–G_8_) were used for crossing experiments. A total of nine groups were set up for reciprocal crossing: DT *w*MC‐DT ♀ × *w*MC‐DT♂; ST *w*C‐ST♀ × *w*M‐ST♂; *w*M‐ST♀ × *w*C‐ST♀; *w*M‐ST♀ × *w*MC‐DT♂; *w*C‐ST♀ × *w*MC‐DT♂; wild‐type WT♀ × *w*M‐ST♀; WT♀ × *w*C‐ST♂, and WT♀ × WT♂ (control group). For each group, the mating pairs were confined in a leaf‐clip cage on a cotton plant for 5 days, and the eggs laid on the plant were then placed into a climate incubator for further development till adult emergence (L14:D10 and 65 ± 1% RH at 28°C). The progenies were collected, and the number of off‐spring per female and the percentage of males were calculated. CI level was assessed as the proportion of male offspring. Three biological replicates were performed for each group.

### Genome sequencing and identification of Cif genes in *w*Ccep

The genome of *Wolbachia w*Ccep strain was sequenced by Illumina and PacBio technologies (Ellegaard et al., 2013). For isolation and purification of *Wolbachia w*Ccep, the infected rice moth was allowed to oviposit, and the eggs were collected. The eggs were dechorionated in bleach, rinsed with sterile distilled water, and then homogenised in phosphate‐buffered saline (PBS) buffer with a sterile micropestle. The homogenate was centrifuged at 400 × g for 5 min, and the supernatant was transferred to a new tube and centrifuged at 5400 × g for 5 min. The pellet was resuspended in PBS and then centrifuged at 400 × g for 5 min to remove remaining debris. The supernatant was slowly pushed through a 5 μm pore size filter (Millipore, Bedford, MA) with a syringe, followed by a 2.7 μm pore size filter (Whatman, USA). The filtrate was centrifuged at 6900 × g for 15 min. After decanting the supernatant, a bacterial pellet (*Wolbachia* cells) was obtained. The purified *Wolbachia* was confirmed by PCR using the primers 81F/522R targeting *wsp* of Supergroup B *Wolbachia* (Zhou et al., 1998), and then quickly ground and transferred to a preheated 50‐mL centrifuge tube containing 15 mL CTAB solution, which was mixed and placed in a constant‐temperature water bath at 65°C, lysed for 60 min, and mixed once every 15 min. The sample was cooled to room temperature before centrifuged at 5000 rpm for 10 min at room temperature. The supernatant was added to an equal volume of phenol/chloroform/isoamyl alcohol and centrifuged. The upper liquid was extracted one more time, and then transferred to a new tube with addition of 2/3 volume of isopropanol and 50 μL 3 M sodium acetate, and placed in a refrigerator (−20°C) overnight for precipitation. The mixture was added with 750 μL ethanol (75%) and centrifuged at 5000 rpm for 5 min. This step was repeated once. The dried pellet was finally dissolved in Tris‐EDTA buffer (10 mM Tris, 1 mM EDTA, pH 8.0) and frozen at −20°C for genome sequencing on a PacBio RS II and Illumina HiSeq 4000 platform (Illumina Inc., San Diego, CA, USA) by Beijing Genomics Institute (Beijing, China).

The draft genome of *w*Ccep was assembled using the Celera Assembler against a high‐quality corrected circular consensus sequence subreads set. The assembled genome was annotated using Prokka version 1.14 (Seemann, 2014) with default parameters. The Cif genes in the genome of *w*Ccep were searched with TBlastN (https://blast.ncbi.nlm.nih.gov/Blast.cgi) using all *cif* gene sequences available to date.

### Phylogenetic analysis of Cif proteins in *w*Ccep and *w*Mel

The Cif proteins encoded by the *cif* genes identified in the *w*Ccep genome sequenced in this study and retrieved from the *w*Mel genome (GenBank acc. no. GCF_000008025.1) were aligned with reference homologous sequences (Table S2). Phylogenetic analysis was performed using the maximum likelihood algorithm on MEGA7.1. The percentage of replicate trees in which the associated taxa clustered together in the bootstrap test (1000 replicates) are shown at the node. The evolutionary distances were computed using the Poisson correction method. All positions containing gaps and missing data are eliminated.

### Structural comparison of CIF proteins between *w*Ccep and *w*Mel

CIF proteins were preliminarily analysed using the SMART programme (http://smart.embl-heidelberg.de/; Schultz et al., 1998). The proteins were further analysed for the presence of putative domains using the HHpred protein domain prediction tool (https://toolkit.tuebingen.mpg.de/#/tools/hhpred) (Söding et al., 2005; Zimmermann et al., 2018) with default parameters, and annotations with *p* > 80% were recorded (Shropshire et al., 2022). The databases SCOPe70 (v.2.07), Pfam (v.33.1), SMART (v6.0) and COG/KOG (v1.0) (Lindsey et al., 2018; Martinez et al., 2022) were used to confirm SMART‐identified domains and identify additional domain structures.

### Comparison of the tertiary structures of Cif proteins between *w*Ccep and *w*Mel

The tertiary structures of CifA I^*w*Ccep^, CifB I^*w*Ccep^, CifA IV^*w*Ccep^, CifB IV^*w*Ccep^, CifA I^*w*Mel^ and CifB I^*w*Mel^ proteins were generated by AlphaFold2.2.3 as a local server and based on full AlphaFold database (Jumper et al., 2021). The max_template_date was set to ensure that all proteins are generated off the same set of starting templates (last accessed: 21 September 2020). The similarity between tertiary structures was calculated using the Zhanglab TM‐score Server (http://zhanggroup.org/TM-align/; Xu & Zhang, 2010). The structural difference between the CIF proteins of *w*Ccep and *w*Mel was evaluated based on their similarities. AlphaFold‐Multimer (Evans et al., 2021) was used to predict the binding complex of CifA I^*w*Ccep^ – CifB I^*w*Ccep^ full sequences with multiple sequence alignments set as the all genetics database used at CASP14. The prediction of complexes was run fifth with different random seeds and 25 models were obtained. Finally, the complex with the highest quality score (p_lDDT_ = 0.726) was selected. Among the complexes, the tail of CifB I^*w*Ccep^ that contained a DUB domain (residue 753–1107) was removed due to being far away from the core and having a low predicted mass, which was consistent with previous studies (Wang et al., 2022). The remainder contained two PD‐(D/E)XK (pseudo) nuclease domains (residue 1–752) for further optimization with subsequent MD simulations.

### MD simulations

MD simulations were performed by using Desmond programme of Schrödinger 2021‐3 (Bowers et al., 2006) and the OPLS4 (Lu et al., 2021) protein force field. The binding complex of CifA I^*w*Ccep^‐CifB I^*w*Ccep^_2ND_ obtained in the last step was explicitly solvated with TIP3P (Jorgensen et al., 1983) water molecules under cubic periodic boundary conditions for a 15 Å buffer region. The overlapping water molecules were removed and 0.15 M KCl was added, and the systems were neutralised by adding K^+^ as counter ions. The electrostatic interactions were calculated under elastic simulations by Verlet and cg algorithms and particle‐mesh Ewald method, and energy minimization was performed using the steepest descent method for the maximum number of steps (50,000 steps). The Coulomb force cut‐off distance and the van der Waals radius cut‐off distance were both 1.4 nm. The system was equilibrated using a regular system (NVT) and an isothermal isobaric system (NPT), followed by 100 ns MD simulations at constant temperature and pressure. The V‐rescale temperature coupling method was used to control the simulation temperature to 300 K. RMSD was used to observe the local site metastability of the system during the simulation and calculated based on C‐alpha atoms. The plot was presented using PyMOL 2.4.1. The Ramachandran plot of the eventual model was generated with Schrödinger 2021‐3.

### Data analysis

Statistical differences were analysed using One‐way analysis of variance followed by SNK test and Tukey's post hoc tests (*p* < 0.05) on SPSS v.27.0 software (SPSS Inc., Chicago, IL, USA).

## AUTHOR CONTRIBUTIONS

**Jing Li:** Investigation (lead); methodology (lead); writing – original draft (lead). **Bei Dong:** Data curation (equal); investigation (equal). **Yong Zhong:** Formal analysis (equal); methodology (equal). **Zheng‐Xi Li:** Conceptualization (lead); funding acquisition (lead); methodology (lead); project administration (lead); supervision (lead); writing – review and editing (lead).

## CONFLICT OF INTEREST STATEMENT

The authors declare no conflicts of interest.

## Supporting information


**Data S1:** Supporting information.

## Data Availability

The data that support the findings of this study are openly available in GenBank at https://ncbi.nlm.nih.gov (acc.no. CP087954).
