# Supplementary material for: Transinfected Wolbachia strains induce a complex of cytoplasmic incompatibility phenotypes: Roles of CI factor genes
Source: Environ Microbiol Rep. 2023 May 16;15(5):370–82. doi: 10.1111/1758-2229.13169 (PMC10472523; doi:10.1111/1758-2229.13169)
Supplement: Supplementary file 1 — Data S1: Supporting information. [file EMI4-15-370-s001.docx]

**Transinfected *Wolbachia* strains induce a complex of cytoplasmic incompatibility phenotypes: Roles of CI factor genes**

Jing Li^1^, Bei Dong^2^, Yong Zhong^3^, Zheng-Xi Li^1†^

^1^ Department of Entomology and Key Laboratory of Pest Monitoring and Green Management, MOA, College of Plant Protection, China Agricultural University, 2 Yuanmingyuan West Road, Beijing 100193, China.

^2^ Jinan Academy of Agricultural Sciences, No.717, Mingfa Road, Changqing District, Jinan 250316, Shandong, China.

^3^ Pingxiang Customs Comprehensive Technical Service Center, Pingxiang 532600, Guangxi, China.

^†^Author for correspondence: Zheng-Xi Li, Department of Entomology, China Agricultural University, 2 Yuanmingyuan West Road, Beijing 100193, China.

E-mail: [zxli@cau.edu.cn](mailto:zxli@cau.edu.cn).


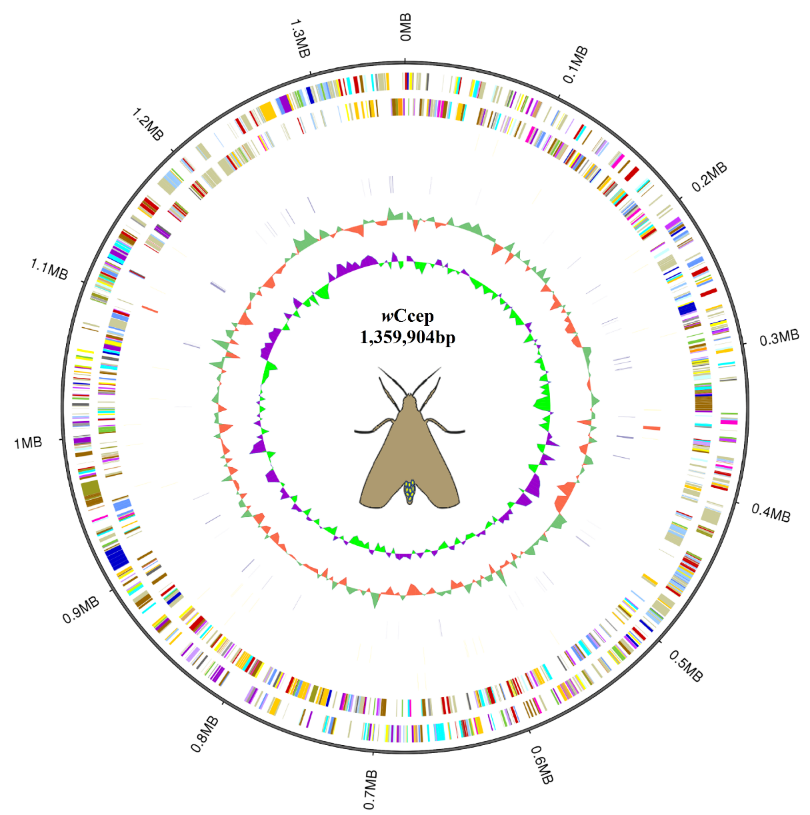


**Figure S1.** Circular map of the genome of *Wolbachia* *w*Ccep strain


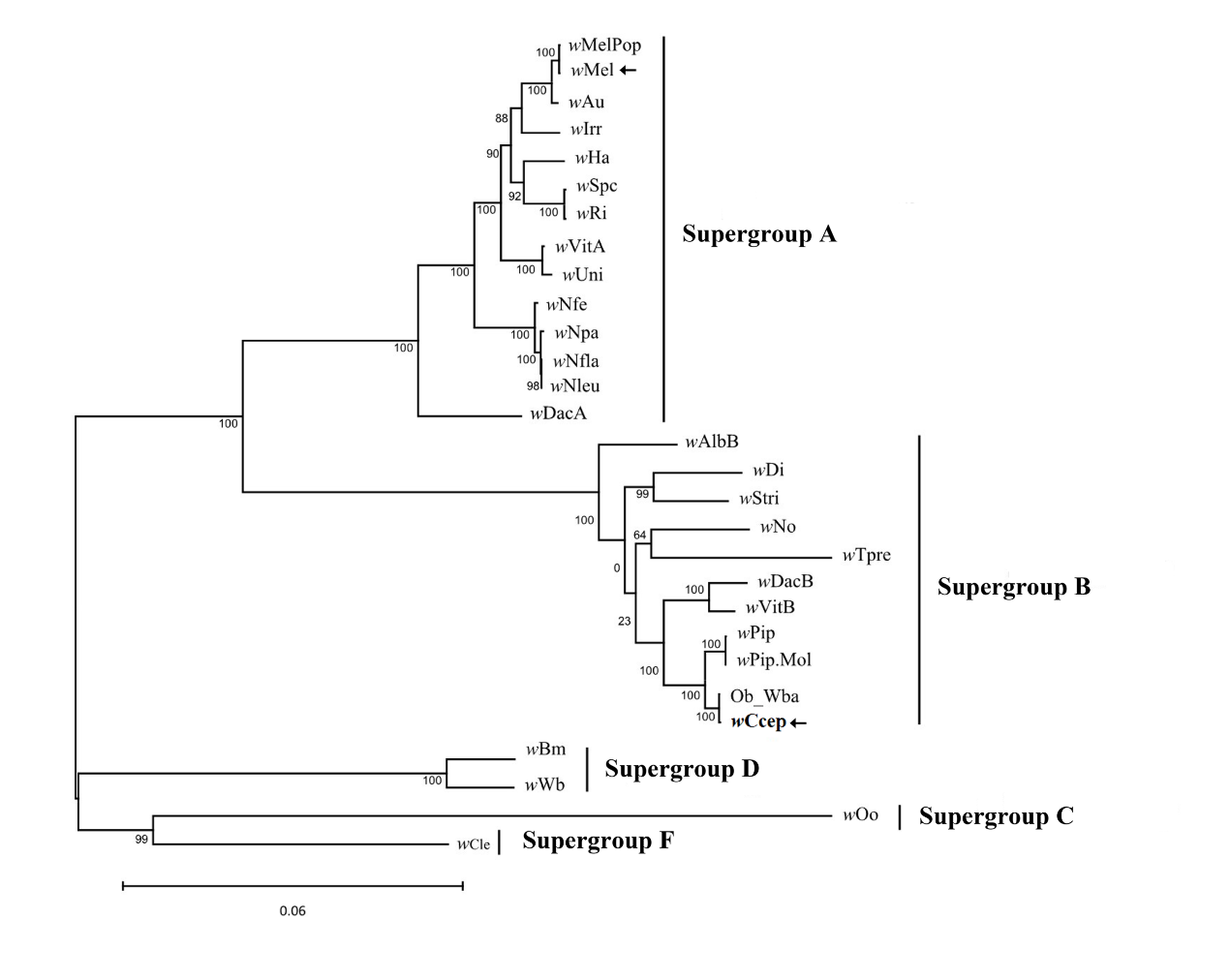


**Figure S2.** A maximum likelihood (ML) phylogenetic tree of *Wolbachia* strains calculated using concatenated protein sequences of 29 single-copy genes (84,369 amino acids). The tree (bootstrap n=1,000; Poisson correction; complete gap deletion) is constructed based on a ClustalW alignment of protein sequences. The scale bar represents the average number of substitutions per site.

**Table S1** *Wolbachia* genome sequences used in phylogenetic analysis

| *Wolbachia* strain | *S*upergroup | Native host | Phenotype*^a^* | Genome | Size (Mb) | GC% | Gene | Protein | Coding density % | RefSeq acc. no. |
| --- | --- | --- | --- | --- | --- | --- | --- | --- | --- | --- |
| *w*Au | A | *Drosophila simulans* | CI | Complete | 1.27 | 35.2 | 1265 | 1099 | 86.88 | GCF_000953315.1 |
| *w*Uni | A | *Muscidifurax uniraptor* | PI | Draft | 0.87 | 35.1 | 912 | 827 | 90.68 | GCF_000174095.1 |
| *w*Nfe | A | *Nomada ferruginata* |  | Draft | 1.34 | 35.2 | 1364 | 1078 | 79.03 | GCF_001675785.1 |
| *w*Npa | A | *Nomada panzeri* |  | Draft | 1.34 | 35.2 | 1372 | 1102 | 80.32 | GCF_001675775.1 |
| *w*Nfla | A | *Nomada flava* |  | Draft | 1.33 | 35.2 | 1354 | 1090 | 80.50 | GCF_001675695.1 |
| *w*Nleu | A | *Nomada leucophthalma* |  | Draft | 1.37 | 35.2 | 1382 | 1123 | 81.26 | GCF_001675715.1 |
| *w*DacA | A | *Dactylopius coccus* |  | Draft | 1.17 | 35.1 | 1220 | 984 | 80.66 | GCF_001648025.1 |
| *w*Ha | A | *Drosophila simulans* | CI | Complete | 1.30 | 35.1 | 1263 | 1126 | 89.15 | GCF_000376605.1 |
| ***w*Mel** | **A** | ***Drosophila melanogaster*** | **CI** | **Complete** | **1.27** | **35.2** | **1270** | **1100** | **86.61** | **GCF_000008025.1** |
| *w*MelPop | A | *Drosophila melanogaster* | CI | Draft | 1.24 | 35.6 | 1165 | 1029 | 88.33 | GCF_000475015.1 |
| *w*Ri | A | *Drosophila simulans* | CI | Complete | 1.45 | 35.2 | 1403 | 1254 | 89.38 | GCF_000022285.1 |
| *w*Rec | A | *Drosophila recens* | CI + MK | Draft | 1.13 | 35.2 | 1132 | 965 | 85.25 | GCF_000742435.1 |
| *w*VitA | A | *Nasonia vitripennis* | CI | Draft | 1.21 | 34.9 | 1150 | 1042 | 90.61 | GCA_001983615.1 |
| *w*Spc | A | *Drosophila subpulchrella* |  | Draft | 1.42 | 35.7 | 1438 | 1239 | 86.16 | GCF_002300525.1 |
| *w*Irr | A | *Haematobia irritans* |  | Complete | 1.35 | 35.3 | 1419 | 1229 |  | GCA_009732755.1 |
| *w*DacB | B | *Dactylopius coccus* |  | Draft | 1.50 | 34.0 | 1530 | 1041 | 68.04 | GCF_001648015.1 |
| *w*Di | B | *Diaphorina citri* | Undetermined | Draft | 0..75 | 34.0 | 688 | 617 | 89.68 | GCF_000331595.1 |
| *w*AlbB | B | *Aedes albopictus* | CI | Draft | 1.16 | 33.8 | 1110 | 955 | 86.04 | GCF_000242415.2 |
| *w*No | B | *Drosophila simulans* | CI | Complete | 1.30 | 34.0 | 1231 | 1065 | 86.52 | GCF_000376585.1 |
| *w*PipMol | B | *Culex pipiens complex* | CI | Draft | 1.54 | 34.2 | 1484 | 1286 | 86.66 | GCF_000156735.1 |
| *w*Pip | B | *Culex pipiens complex* | CI | Complete | 1.48 | 34.2 | 1402 | 1257 | 89.66 | GCF_000073005.1 |
| *w*Stri | B | *Laodelphax striatella* | CI | Draft | 1.23 | 33.8 | 1154 | 1011 | 87.61 | GCF_001637495.1 |
| *w*VitB | B | *Nasonia vitripennis* | CI | Draft | 0.23 | 34.0 | 250 | 150 | 60.00 | GCF_000204545.1 |
| *w*Bol1 | B | *Hypolimnas bolina* | CI + MK | Draft | 1.38 | 33.9 | 1293 | 1139 | 88.09 | GCF_000333775.1 |
| *w*Tpre | B | *Trichogramma pretiosum* | PI | Complete | 1.13 | 33.9 | 1106 | 827 | 74.77 | GCF_001439985.1 |
| Ob_Wba | B | *Operophtera brumata* |  | Draft | 1.12 | 33.8 | 1118 | 952 | 85.15 | GCF_001266585.1 |
| ***w*Ccep** | **B** | ***Corcyra cephalonica*** | **CI** | **Complete** | **1.36** | **34.5** | **1278** | **1190** |  | **GCF_020995475.1** |
| *w*Oo | C | *Onchocerca ochengi* | OM | Complete | 0.96 | 32.1 | 733 | 645 |  | GCF_000306885.1 |
| *w*Bm | D | *Brugia malayi* | OM | Complete | 1.08 | 34.2 | 1044 | 839 |  | GCF_000008385.1 |
| *w*Cle | F | *Cimex lectularius* | OM | Complete | 1.25 | 36.3 | 1246 | 981 | 78.73 | GCF_000829315.1 |

*^a^* CI: cytoplasmic incompatibility; PI: parthenogenesis-inducing; MK: male-killing; OM: obligate mutualism, and Undetermined: phenotype is not assayed.

**Table S2 CIF protein sequences used for phylogenetic analysis**

| *Wolbachia*  strain | Native host | GenBank acc. no. (CifA) | GenBank acc. no. (CifB) |
| --- | --- | --- | --- |
| *w*VitA4 | *Nasonia vitripennis* | ONI58213.1 | ONI58212.1 |
| *w*Sol | *Ceratosolen solmsi* | AGK87106.1 | AGK87078.1 |
| *w*Ha | *Drosophila simulans* | WP_015588933.1 |  |
| *w*DacB | *Dactylopius coccus* | WP_019236549.1 |  |
| *w*Bol1-b | *Hypolimnas bolina* | WP_019236549.1 | WP_019236548.1 |
| *w*Pip | *Culex quinquefasciatus* | WP_012481787.1 |  |
| *w*Spc | *Drosophila subpulchrella* | WP_044471237.1 |  |
| *w*Mel | *Drosophila melanogaster* | WP_010962721.1 | WP_010962722.1 |
| *w*Rec | *Drosophila recens* | WP_038198916.1 |  |
| *w*Ri | *Drosophila simulans* | WP_012673191.1 |  |
| *w*PipJHB | *Culex quinquefasciatus JHB* | WP_007302988.1 |  |
| *w*Pip_Pel | *Culex quinquefasciatus Pel* | 7ESX_A | 7ESY_B |
| *w*MelPop | *Drosophila melanogaster* |  | WP_038228284.1 |
| *w*Ha1 | *Drosophila simulans* |  | AGJ99743.1 |
| *w*SuziB | *Drosophila suzukii* |  | WP_044471243.1 |
| *w*DacB | *Dactylopius coccus* |  | OAM06074.1 |
| *w*No | *Drosophila simulans* |  | WP_015588932.1 |
| *w*Ccep1 | *Corcyra cephalonica* | OP767524 | OL539522 |
| *w*Ri | *Drosophila simulans* | WP_012673228.1 | WP_012673227.1 |
| *w*Ana_India | *Drosophila ananassae* | WP_012673228.1 |  |
| *w*Suz | *Drosophila suzukii* | WP_044471252.1 | WP_044471251.1 |
| *w*AblB | *Aedes albopictus* | WP_006014162.1 | WP_006014164.1 |
| *w*VitA | *Nasonia vitripennis* | WP_077188281.1 | WP_077188282.1 |
| *w*Uni | *Muscidifurax uniraptor* | WP_077190377.1 | WP_077188282.1 |
| *w*No | *Drosophila simulans* | WP_015587806.1 | WP_015587805.1 |
| *w*Ana | *Drosophila ananassae* |  | WP_015587805.1 |
| *w*AlbB | *Aedes albopictus* | WP_006012794.1 | WP_006012795.1 |
| *w*Pip | *Culex pipiens complex* | WP_007302980.1 | WP_182367238.1 |
| *w*Pip_Mol | *Culex pipiens complex* | WP_007302980.1 | WP_007302979.1 |
| *w*PipJHB | *Culex quinquefasciatus JHB* | WP_007302980.1 |  |
| *w*Pip_Pel | *Culex quinquefasciatus Pel* | 7ESZ_B | 7ESZ_A |
| *w*Bol1-b2 | *Hypolimnas bolina* | WP_019236479.1 |  |
| *w*Ccep2 | *Corcyra cephalonica* | OP767525 | OP767526 |
| *w*Ha | *Drosophila simulans* |  | WP_019236480.1 |
| *w*DacB | *Dactylopius coccus* | WP_064085535.1 | WP_064085536.1 |
| *w*Stri | *Laodelphax striatellus* | WP_063631187.1 | WP_063631188.1 |
| *w*Stri | *Laodelphax striatellus* | WP_063631193.1 |  |


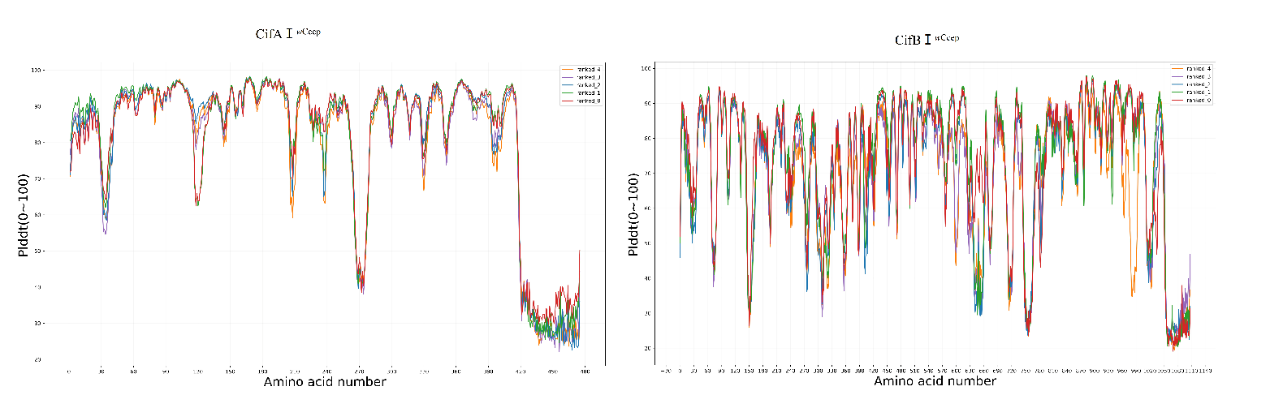


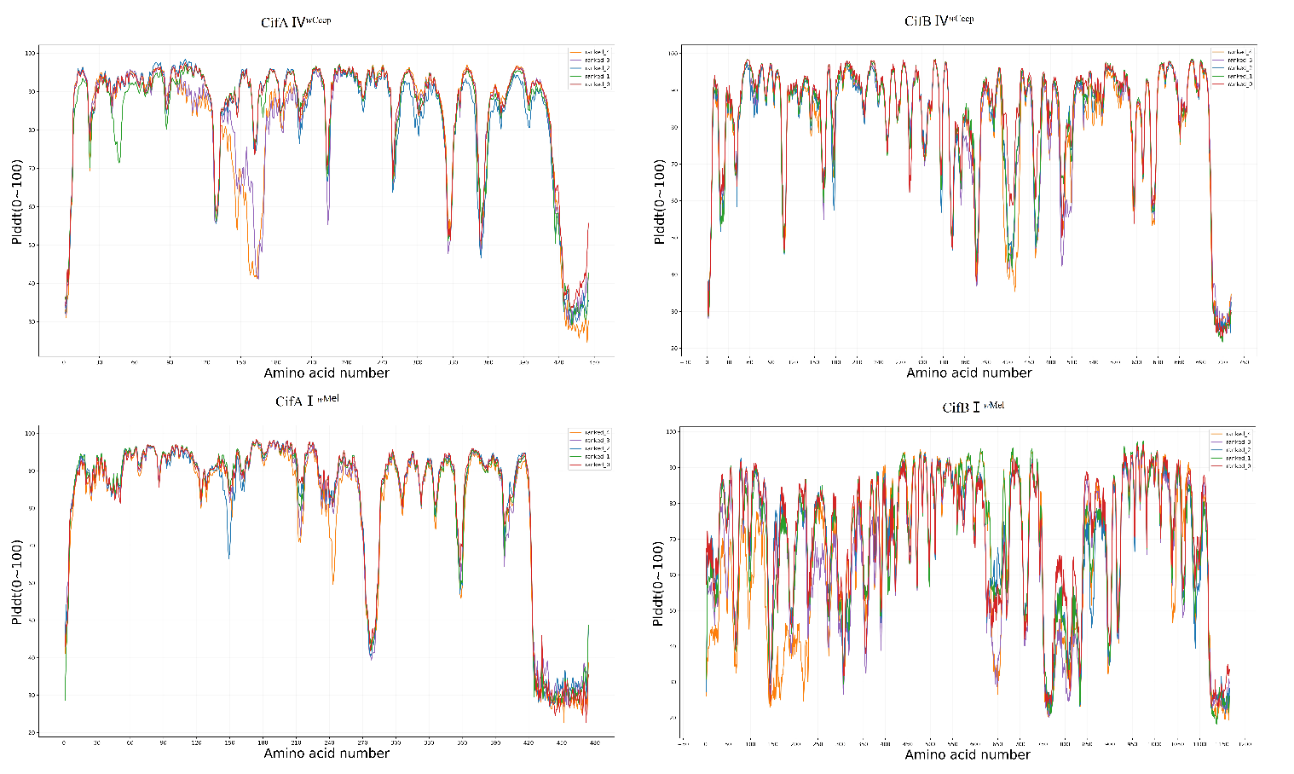


**Figure S3. Alphafold structural confidence for CIF proteins.** Alphafold structural confidence per amino acid position in the protein. Confidence is displayed as lDDT, where higher values represent higher confidence. Five models are produced for each protein. All five models are displayed here and are numbered in order of confidence. The model with the highest confidence for each protein (ranked_ 0) is used for all other analyses.


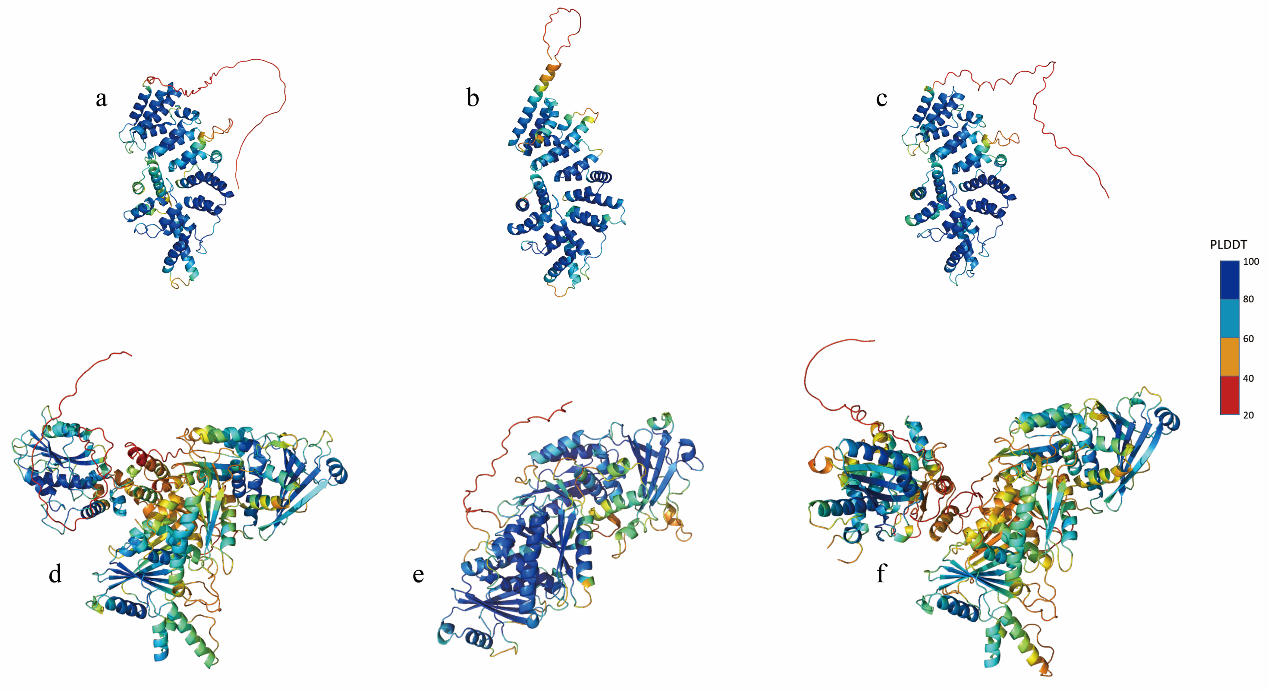


**Figure S4.** Tertiary structures of CIF proteins of *w*Ccep and *w*Mel generated by AlphaFold. a. CifA Ⅰ*^w^*^Ccep^; b. CifA Ⅳ*^w^*^Ccep^; c. CifA*^w^*^Mel^; d. CifB Ⅰ*^w^*^Ccep^; e. CifB Ⅳ*^w^*^Ccep^; f. CifB*^w^*^Mel^.


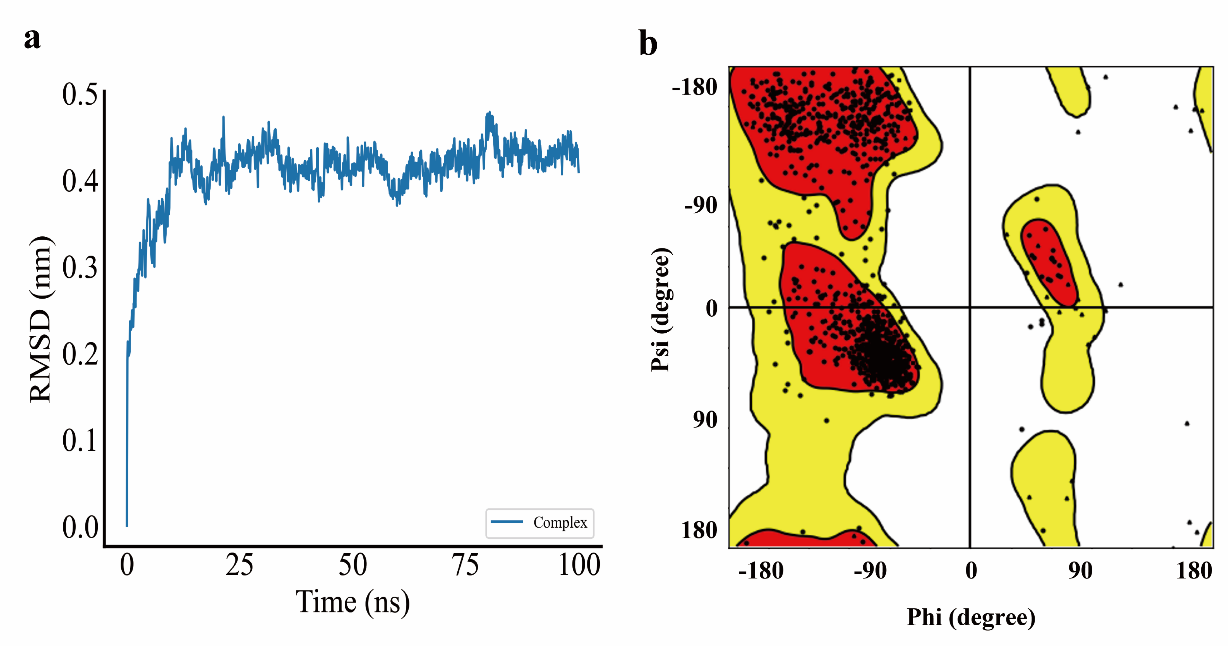


**Figure S5.** Molecular dynamic simulation and validation of the model of the CifA*^w^*^Ccep^ - CifB*^w^*^Ccep^ complex. (a) RMSD of CifA*^w^*^Ccep^ - CifB*^w^*^Ccep^ complex along the simulation trajectory. (b) Ramachandran plot of the optimized CifA*^w^*^Ccep^ - CifB*^w^*^Ccep^ binding complex.
